# Plasma Proteomics Elucidated a Protein Signature in COVID-19 Patients with Comorbidities and Early-Diagnosis Biomarkers

**DOI:** 10.3390/biomedicines12040840

**Published:** 2024-04-10

**Authors:** Víctor Urbiola-Salvador, Suiane Lima de Souza, Katarzyna Macur, Paulina Czaplewska, Zhi Chen

**Affiliations:** 1 Intercollegiate Faculty of Biotechnology of University of Gdańsk and Medical University of Gdańsk, University of Gdańsk, 80-307 Gdańsk, Poland; v.urbiolasalvador.373@studms.ug.edu.pl; 2 Faculty of Biochemistry and Molecular Medicine, University of Oulu, 90220 Oulu, Finland; suiane.limads@gmail.com; 3 Laboratory of Mass Spectrometry-Core Facility Laboratories, Intercollegiate Faculty of Biotechnology University of Gdańsk and Medical University of Gdańsk, University of Gdańsk, 80-309 Gdańsk, Poland; katarzyna.macur@biotech.ug.edu.pl (K.M.); paulina.czaplewska@ug.edu.pl (P.C.)

**Keywords:** SARS-CoV-2, COVID-19, plasma proteomics, LC-MS/MS, biomarker, inflammation, complement, comorbidity, acute-phase reaction, diagnosis

## Abstract

Despite great scientific efforts, deep understanding of coronavirus-19 disease (COVID-19) immunopathology and clinical biomarkers remains a challenge. Pre-existing comorbidities increase the mortality rate and aggravate the exacerbated immune response against the severe acute respiratory syndrome coronavirus-2 (SARS-CoV-2) infection, which can result in more severe symptoms as well as long-COVID and post-COVID complications. In this study, we applied proteomics analysis of plasma samples from 28 patients with SARS-CoV-2, with and without pre-existing comorbidities, as well as their corresponding controls to determine the systemic protein changes caused by the SARS-CoV-2 infection. As a result, the protein signature shared amongst COVID-19 patients with comorbidities was revealed to be characterized by alterations in the coagulation and complement pathways, acute-phase response proteins, tissue damage and remodeling, as well as cholesterol metabolism. These altered proteins may play a relevant role in COVID-19 pathophysiology. Moreover, several novel potential biomarkers for early diagnosis of the SARS-CoV-2 infection were detected, such as increased levels of keratin K22E, extracellular matrix protein-1 (ECM1), and acute-phase response protein α-2-antiplasmin (A2AP). Importantly, elevated A2AP may contribute to persistent clotting complications associated with the long-COVID syndrome in patients with comorbidities. This study provides new insights into COVID-19 pathogenesis and proposes novel potential biomarkers for early diagnosis that could be facilitated for clinical application by further validation studies.

## 1. Introduction

Since the coronavirus-19 disease (COVID-19) outbreak, extensive research efforts have improved our understanding of the severe acute respiratory syndrome coronavirus 2 (SARS-CoV-2) infection pathogenesis, as well as diagnostics, treatment, and prevention with the effective design of vaccines [1]. The pathophysiology of the SARS-CoV-2 infection is closely interconnected with the immune response, resulting in diverse clinical presentations from asymptomatic/mild to severe with high mortality rates. In fact, the SARS-CoV-2-induced tissue damage recruits immune cells, causing a local and systemic inflammatory response, also called a cytokine storm, which in severe cases leads to pneumonia, microthrombi deposition, systemic symptoms, and multi-organ failure in fatal cases. With the evolution of the virus and the emergence of new variants, continuous research must characterize the interaction between the SARS-CoV-2 infection and the immune response [2].

The heterogeneity in the COVID-19 response is caused by clinical variabilities such as sex, age, and ethnicity, as well as pre-existing comorbidities. Cardiovascular diseases, cancer, diabetes, and obesity, among others, are linked with higher severity and mortality rates [3]. Despite great advances in COVID-19 research, a deeper understanding of COVID-19 immunopathology, especially in patients with pre-existing comorbidities, and the determination of specific COVID-19 biomarkers, is urgently needed [4]. Importantly, proteomics approaches can provide novel insights into the protein changes caused by the SARS-CoV-2 infection, at the cellular and systemic levels, to identify the drivers of the pathogenesis [5]. In fact, the proteomics characterization of COVID-19 patients’ plasma is a non-invasive strategy that can reflect the disease status and determine potential biomarkers associated with the pathophysiological mechanisms of the SARS-CoV-2 infection.

In this study, we applied tandem mass spectrometry coupled with liquid chromatography (LC-MS/MS) proteomics analysis to plasma samples obtained from the Finnish Clinical Biobank. The samples consist of 28 patients with SARS-CoV-2, 19 of whom were patients with pre-existing comorbidities (CPs) and their age- and sex-matched healthy controls (HCs) and comorbidity disease controls (DCs). The aim of this study was to characterize the plasma protein changes associated with the presence or absence of comorbidities and the time of infection. We found a common protein signature among COVID-19 patients with comorbidities characterized by protein alterations involved in the coagulation and complement pathways, tissue damage and remodeling, acute-phase reaction, as well as cholesterol metabolism, among others. Moreover, novel potential diagnostic biomarkers of early SARS-CoV-2 infection were detected, including the protein keratin K22E, extracellular matrix protein 1 (ECM1), and acute-phase response protein α-2-antiplasmin (A2AP). This study determined novel insights into the plasma protein changes caused by the SARS-CoV-2 infection that may lead to an application of these potential biomarkers after further studies.

## 2. Materials and Methods

### 2.1. Study Cohort

This retrospective study included 28 plasma samples collected from patients with the SARS-CoV-2 virus (25% males, age range 19–79). Nineteen of these were CPs with other pre-existing diseases, while the remaining nine patients were without comorbidities. Patients without comorbidities were age- and sex-matched to SARS-CoV-2 virus-negative healthy subjects without any diseases. The 19 CPs were age- and sex-matched with 19 healthy controls and a total of 20 DCs suffering from the same disease(s). Among the 19 CPs, two CPs were age- and sex-matched to two disease controls with the same clinical conditions, while for another patient, a disease control was not found. (Table S1). Plasma samples and clinical information were obtained from the Finnish Clinical Biobank, Tampere. The study was conducted in accordance with the Declaration of Helsinki and was approved by the HUS ethics committee. All participants provided informed consent.

### 2.2. Sample Preparation for Mass Spectrometry

Proteins were extracted from plasma samples with lysis buffer containing 1% SDS and 50 mM DTT in 100 mM Tris-HCl, pH 8.0, with protease and phosphatase inhibitors. Samples were incubated at 95 °C for 10 min, and protein concentrations were determined in NanoDrop 2000 at 280 nm. Samples were processed according to the Filter Aided Sample Preparation protocol [6]. Briefly, 100 μg of proteins were transferred to Microcon 10 kDa filters (Merck, Rahway, NJ, USA) and were washed three times with 200 µL of urea buffer (8 M urea in 100 mM Tris-HCl, pH 8.5) at 10,000 RCF for 20 min at room temperature. Free cysteines were alkylated by incubation in the darkness for 20 min at room temperature with 100 µL of 55 mM iodoacetamide in urea buffer. Samples were centrifuged at 10,000 RCF for 15 min, followed by three washes with 100 µL of urea and two final washes with 100 µL of digestion buffer (50 mM Tris-HCl, pH 8.5). After washing steps, proteins were digested by incubation at 37 °C overnight with Sequencing Grade Modified Trypsin (Promega, Madison, WI, USA) at a trypsin–protein ratio of 1:100 in 60 µL of digestion buffer. Peptides were eluted in a new collection tube by centrifugation and with two additional elutions with 125 and 100 µL of digestion buffer. Next, trypsin activity was quenched with a final concentration of 0.1% trifluoroacetic acid. Peptide concentrations were measured in Nanodrop at 280 nm, and 10 μg of peptides were desalted via the STop And Go Extraction (STAGE) Tips protocol [7] using Empore C18 extraction disks (CDS Analytical LLC, Oxford, PA, USA) with elution by 60% acetonitrile/1% acetic acid solution. Samples were dried using SpeedVac and stored at −20 °C until analysis.

### 2.3. Mass Spectrometry Analysis

The LC-MS/MS analysis was performed in the positive ion mode using a TripleTOF 5600+ mass spectrometer equipped with TurboV Ion Source (SCIEX, Framingham, MA, USA) and coupled with the EkspertMicroLC 200 Plus System (Eksigent, Redwood City, CA, USA). SCIEX Analyst TF 1.8.1 software (SCIEX, Framingham, MA, USA) controlled the microLC-MS/MS system. Moreover, 2 µg of peptides were injected per technical replicate, and the chromatographic separations were performed with a 5 µL/min flow for 60 min on a ChromXP C18CL column (3 μm, 120 Å, 150 × 0.3 mm) (Eksigent, Redwood City, CA, USA) placed in a column oven at 35 °C. Peptides were separated with a gradient from 11% to 35% of acetonitrile in 0.1% formic acid. The mass spectrometer operated in data-dependent acquisition mode, and the m/z range of 400–1200 Da was applied for the TOF MS survey scan with an accumulation time of 250 ms. A maximum of top 20 precursor ions with charges between +2 and +5 were selected for collision-induced dissociation (CID) fragmentation with rolling collection energy. Precursor ions were excluded from reselection for 5 s after two occurrences. The product ion spectra were acquired in the range of 100–1800 Da within an accumulation time of 50 ms.

### 2.4. Mass Spectrometry Data Analysis

Acquired raw files were converted with MSConvertGUI 3.0 (ProteoWizard, Palo Alto, CA, USA) to mzML format to use as input for protein identification and quantification analysis using Peaks Studio Xpro 10.6 software (Bioinformatics Solutions Inc., Waterloo, ON, Canada). Peptide sequences were searched against the *Homo sapiens* UniProtKB/Swiss-Prot database (release 2022_03) for peptides with specific trypsin digestion and a maximum of 3 missed cleavages per peptide. Carbamidomethylation was set as a fixed post-translational modification (PTM), whereas N-terminal acetylation and methionine oxidation were set as variable PTMs. Peptides and proteins were identified with a <1% false discovery rate (FDR), and proteins were considered identified with at least 1 significant unique peptide. Label-free quantification was performed based on the integration of the area under the curve (AUC) of peptides with the use of the label-free quantification feature available in PeaksStudio Xpro 10.6 software.

### 2.5. Proteomics Data and Statistical Analysis

Statistical analysis was performed in RStudio (version 1.3.1093, Posit, Boston, MA, USA) using R (version 4.0.3, The R Foundation for Statistical Computing, Vienna, Austria) [8,9]. Peptide results from PeaksStudio software were used for data preprocessing with the “SummarizedExperiment” (version 1.28.0) and “QFeatures” (version 1.8.0) R packages. Peptides that were only detected twice across all samples were removed. Data preprocessing was performed by logarithmic transformation and quantile normalization. Peptides were aggregated in proteins by robust summarization [10]. Quantification reproducibility among technical replicates was evaluated by Pearson correlation. Between-group differences in protein expression levels were analyzed by means of the general linear model regression approach with an analysis of contrasts using the “emmeans” R package (version 1.6.2.1). First, 19 patients with the SARS-CoV 2 virus and comorbidities were involved in searching for differential protein expression due to virus infection and/or coexistent comorbidities by comparing the protein expression levels in patients with the virus to respective healthy and disease-control subjects. The models used in this analysis comprised the presence of comorbidities as a nested confounding factor. The remaining nine comorbidities-free patients were compared to their age- and sex-matched healthy controls to determine protein expression changes due to the virus infection itself without additional confounders in these models. In the last regression modeling, all 28 SARS-CoV-2 virus-positive patients’ samples were analyzed together to search for differential protein expression due to infection time including the presence of comorbidities as a confounding factor. The FDR in contrast analyses was controlled by using the Benjamini and Hochberg correction [11]. Proteins were considered differentially expressed with an FDR-adjusted *p*-value < 0.05. Volcano plots were generated with the R package “ggplot2” (version 3.3.5) and the heatmap with the R package “ComplexHeatmap” (version 2.6.2). The KEGG pathway enrichment analysis via active subnetworks from the STRING database was performed with the R package “pathfindR” (version 1.6.3) with FDR correction [12]. No custom code was used in this analysis.

## 3. Results

### 3.1. Proteomic Profiles of Plasma from COVID-19 Patients and Their Controls

**Figure 1:**
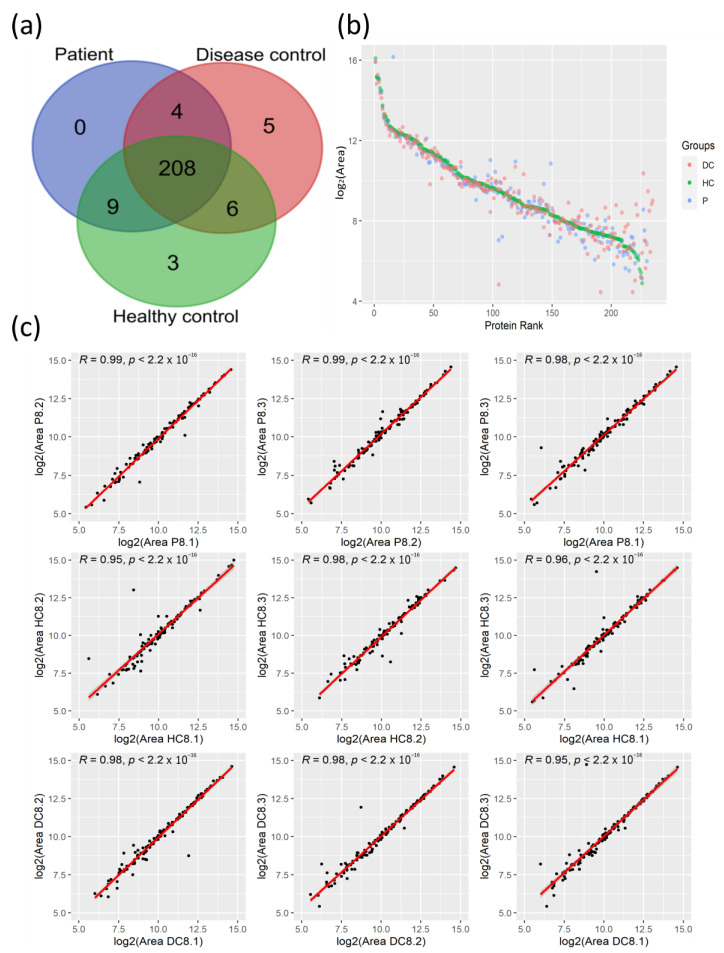
Quantification of plasma proteins from COVID-19 patients and their controls by tandem mass spectrometry coupled with liquid chromatography (LC-MS/MS). (**a**) Venn diagram with identified proteins in the three clinical groups. (**b**) Protein rank plot with the mean of the areas of the 235 identified proteins from each clinical group. (**c**) Correlation analysis of the protein quantified areas (after log_2_ transformation) of three technical replicates from a representative patient and the corresponding controls with the R coefficients and *p*-values. DC, Disease Control; HC; Healthy Control; P, Patient.

We applied the LC-MS/MS proteomics analysis of plasma samples from 28 COVID-19 patients and their corresponding age- and sex-matched HCs to elucidate the protein changes associated with the SARS-CoV-2 infection at the systemic level. As 19 of these patients presented pre-existing comorbidities, the so-called CPs, including cancer, type 2 diabetes mellitus, asthma, type 1 diabetes, and other autoimmune diseases such as rheumatoid arthritis and psoriasis among others, we also analyzed plasma samples from 20 age- and sex-matched DCs with the same pathological conditions. With this experimental design, this study aims to identify common protein changes among SARS-CoV-2 patients with comorbidities. Across the plasma samples from this cohort of 76 individuals, 235 circulating proteins were quantified with an FDR < 1%, from which 208 proteins were commonly identified among the three clinical groups (Figure 1a).

The high-resolution LC-MS/MS mass spectrometry analysis allowed us to quantify proteins that span a concentration range of nine orders of magnitude, as reflected in the high level of coverage of quantified areas in the protein rank plot (Figure 1b). The plasma protein levels of highly abundant proteins were similar between clinical groups, while proteins with lower quantification levels showed high variation among these groups. To evaluate the reproducibility of LC-MS/MS measurements, correlation analysis for randomly selected matched samples was used to demonstrate the high reproducibility among the technical replicates with high correlation coefficients (Figure 1c).

Taken together, the LC-MS/MS analysis of plasma from patients with SARS-CoV-2 and their corresponding controls allowed for the quantification of 235 proteins within a high range of concentrations with high reproducibility among technical replicates.

### 3.2. COVID-19 Patients with Comorbidities Share a Common Plasma Protein Signature

**Figure 2:**
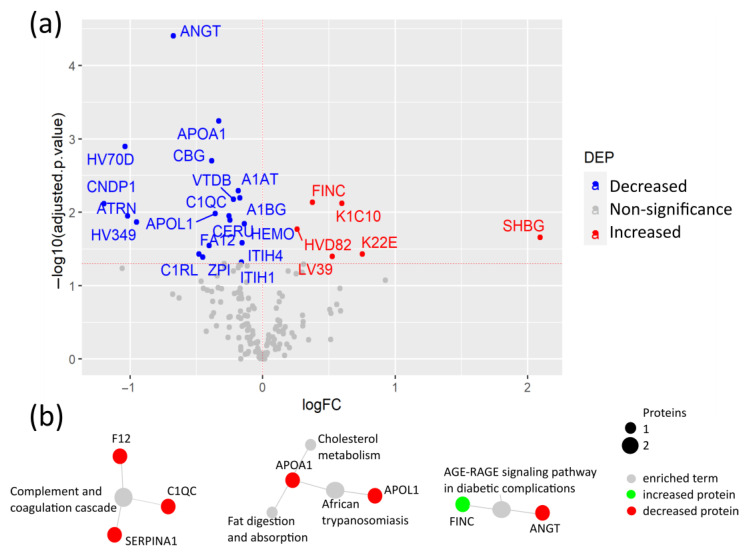
Plasma protein changes in COVID-19 patients with comorbidities compared to their healthy controls. (**a**) Volcano plot of differential expression analysis between COVID-19 patients with comorbidities and age- and sex-matched healthy controls; (**b**) Network of selected pathways from the differentially expressed proteins (DEPs) with KEGG pathway enrichment analysis via active subnetworks from STRING database.

Firstly, to investigate proteomic alterations caused by the SARS-CoV-2 infection under pre-existing pathological conditions, plasma protein levels between the SARS-CoV-2 infected CPs and their age- and sex-matched HCs were compared. Among the 235 quantified proteins, the levels of 25 proteins were significantly changed, including elevated levels of fibronectin (FINC), keratins K1C10 and K22E, sex hormone binding globulin (SHBG), and immunoglobulin variable chains HVD82 and LV39 in CPs (Figure 2a, Table S2). The elevated level of FINC, a mediator of blood clotting, was previously reported, suggesting the reliability of our results [13]. As a key extracellular matrix (ECM) component, FINC can also be considered as an indicator of tissue remodeling after damage caused by the SARS-CoV-2 infection [14]. Moreover, for the first time, increased levels of the two cytoskeletal keratins, K1C10 and K22E, were revealed, which may indicate the SARS-CoV-2-induced damage to epithelial cells.

Proteins with lower plasma levels in CPs compared to HCs included angiotensin II (ANGT), apolipoproteins (APO) A1 and APOL1, vitamin D-binding protein (VTDB), the protease inhibitors alpha-1-antitrypsin (A1AT) and protein Z-dependent protease inhibitor (ZPI), corticosteroid-binding globulin (CBG), alpha-1B-glycoprotein (A1BG), and ceruloplasmin (CERU), among others. Importantly, ANGT showed the most significant change. ANGT is a regulator of blood pressure and cardiac function that is proteolyzed by angiotensin-converting enzyme 2 (ACE2), the receptor of SARS-CoV-2. The reduced levels of ANGT may be caused by the alteration of ACE2 due to the SARS-CoV-2 binding [15]. Noteworthily, two acute-phase reactants, CBG and ZPI, showed lower levels in patients in the COVID-19 studies. Both proteins play a role in reducing the harmful effects of inflammation [16,17]. The reduced plasma levels of CBG in COVID-19 patients have not been previously reported.

The KEGG pathway enrichment analysis of DEPs revealed that the SARS-CoV-2 infection altered the complement and coagulation pathways, cholesterol and fat metabolism, including APOA1 and APOL1, and the AGE-RAGE signaling pathway (Figure 2b, Table S3). APOA1 has anti-viral activity and was previously reported as a strong predictive factor of COVID-19 severity when detected at low levels in COVID-19 patients [18,19]. Moreover, the AGE-RAGE signaling pathway plays a key role in pulmonary inflammatory responses, including viral infection, as well as in diabetes through NF-κB activation and pro-inflammatory cytokine release [20,21].

**Figure 3:**
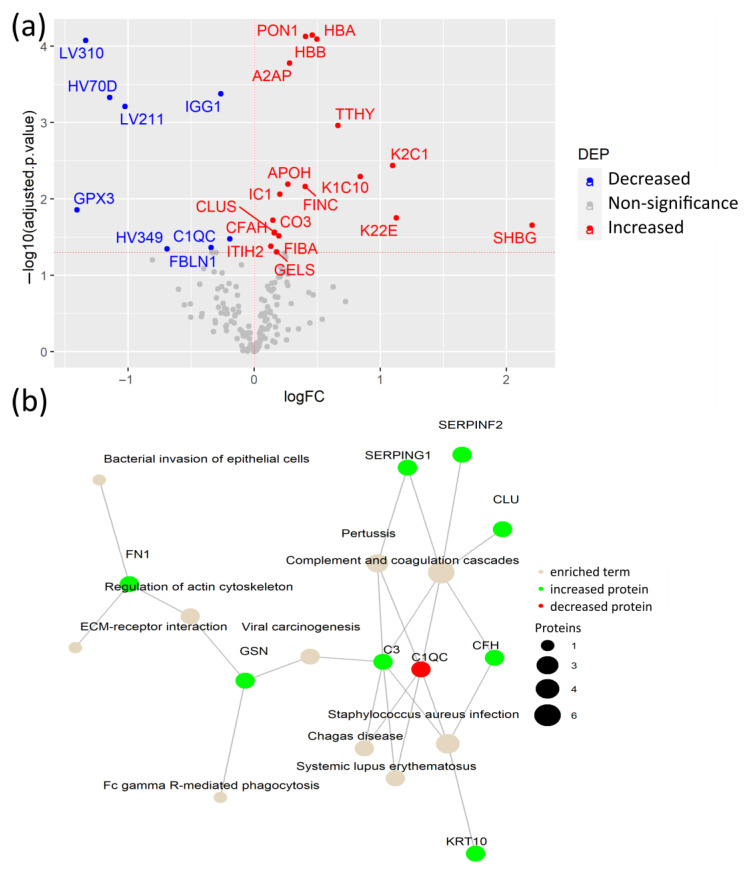
Plasma protein changes caused by SARS-CoV-2 infection in patients with comorbidities. (**a**) Volcano plot of differential expression analysis between COVID-19 patients with comorbidities and age- and sex-matched disease controls; (**b**) Network of KEGG pathway enrichment analysis of DEPs via active subnetworks using STRING database.

To identify specific plasma proteins that are differentially regulated due to the SARS-CoV-2 infection itself in COVID-19 patients with pre-existing comorbidities, we compared CPs versus their DCs. From the 26 detected DEPs, 18 were increased in CPs, including hemoglobin subunits α and β (HBA, HBB), paraoxonase-1 (PON1), and A2AP, among others. The remaining 8 DEPs were decreased in CPs, including the antioxidant enzyme glutathione peroxidase 3 (GPX3), immunoglobulin IGG1, and variable immunoglobulin chains LV310, LV211, HV349, and HC70D (Figure 3a, Table S4). Importantly, this comparison confirmed that increased FINC, together with the keratins K1C10, K22E, and additional K2C1, not detected in the previous comparison, are indicators of tissue damage caused by the SARS-CoV-2 infection rather than from the underlying comorbidities. Moreover, elevated levels of HBA and HBB may have resulted from the hemolysis caused by biochemical modifications within erythrocytes of patients with SARS-CoV-2. SARS-CoV-2 may interact with HBA and HBB, which causes changes in their conformation [22]. Another elevated protein in CPs is PON1, which has antioxidant activity through the hydrolysis of lipoperoxides, participating in the innate immune response to infections and oxidative stress [23]. Of particular interest is the substantial increase in the acute phase inflammatory protein A2AP, which is a part of the plasmin-antiplasmin system that plays a key role in blood coagulation and fibrinolysis [24].

The network of KEGG-enriched pathways in DEPs in CPs, compared to their DCs, showed that elevated levels of proteins in CPs were involved in complement and coagulation cascades, viral carcinogenesis, and ECM-receptor interaction, among others (Figure 3b, Table S5). In fact, the complement system is a fundamental player in the anti-viral innate immune response, including the response against SARS-CoV-2, but its hyperactivation also contributes to the exacerbated immune response in severe COVID-19 cases, suggesting a dual role in the SARS-CoV-2 infection [25]. Among them, the cleavage of C3 (protein CO3) releases C3a anaphylatoxin, which contributes to the hyperinflammatory state of COVID-19 patients [26].

Collectively, COVID-19 patients with different pre-existing comorbidities shared a common plasma protein signature. In addition, the synergistic effect of the SARS-CoV-2 infection and pre-existing comorbidities causes plasma protein changes that are associated with metabolic alterations, coagulation, and innate immune responses.

### 3.3. Coagulation and Cholesterol Metabolism Are Altered in COVID-19 Patients without Comorbidities

**Figure 4:**
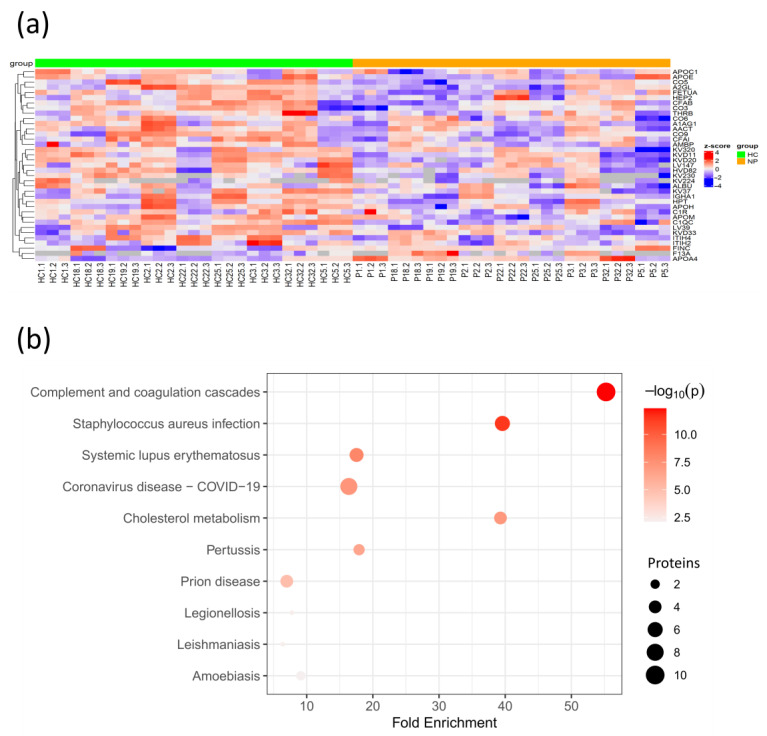
Protein changes in SARS-CoV-2 patients without comorbidities compared to age- and sex-matched healthy controls. (**a**) Heatmap of hierarchical clustering of selected DEPs after z-score normalization. Arrows indicate DEPs highlighted within the text; (**b**) Bubble plot of KEGG enriched terms from DEPs between patients without comorbidities and their healthy controls. NP, non-comorbidity patient; p, *p*-value.

To determine the plasma protein changes in the COVID-19 patients without comorbidities, we quantified and compared plasma protein levels of COVID-19 patients without comorbidities from this cohort to age- and sex-matched HCs. Among the significantly altered protein levels, eight proteins were elevated, while forty were decreased in COVID-19 patients (Figure 4a, Table S6). Among the increased proteins, FINC was also elevated in CPs, suggesting that it is caused by the SARS-CoV-2 infection independently of the pre-existing comorbidities. Meanwhile, the negative acute-phase reactant fetuin-A (FETUA) and APOM were reduced in COVID-19 patients, suggesting that their reduction is caused by the exacerbated innate immune response to the SARS-CoV-2 infection [27,28].

The KEGG pathway enrichment analysis revealed that the DEPs with significant changes were mainly associated with the complement and coagulation cascades and cholesterol metabolism. As expected, the associations with the COVID-19 KEGG and other diseases, such as *Staphylococcus aureus* infection, in which the complement cascade is involved in the innate immune response, were identified (Figure 4b, Table S7). Interestingly, the coagulation factor XIII (F13A), which stabilizes the fibrin clot in the coagulation cascade, was found to be elevated in COVID-19 patients. In fact, coagulopathy plays an essential role in COVID-19 morbidity, and F13A was previously reported to be elevated in COVID-19 patients with less severe symptoms, indicating its capacity to identify COVID-19 patients with mild symptoms [29]. The majority of the proteins associated with cholesterol metabolism, including APOC1, APOH, and APOE, were found to be decreased, except for APOA4, which was elevated in COVID-19 patients. This is supported by a previous study that demonstrated its elevation in COVID-19 patients with mild symptoms as opposed to severe patients [30]. Meanwhile, APOE reduction may be caused by the SARS-CoV-2 infection as APOE can inhibit the SARS-CoV-2 cell entry and reduce inflammation. These findings suggest that cholesterol dysregulation is extended at the systemic level in the SARS-CoV-2 infection. Although the molecular mechanisms of this dysregulation are not yet determined, studies in other infections suggest that cytokines are involved in lipid metabolism alterations [31].

Taken together, the SARS-CoV-2 infection in patients without comorbidities causes the alteration of proteins related to coagulation and cholesterol metabolism, including negative acute-phase reactants that could counteract the innate immune response against the virus.

### 3.4. Early SARS-CoV-2 Infection Is Associated with Immune Protein Changes and Tissue Remodeling

**Figure 5:**
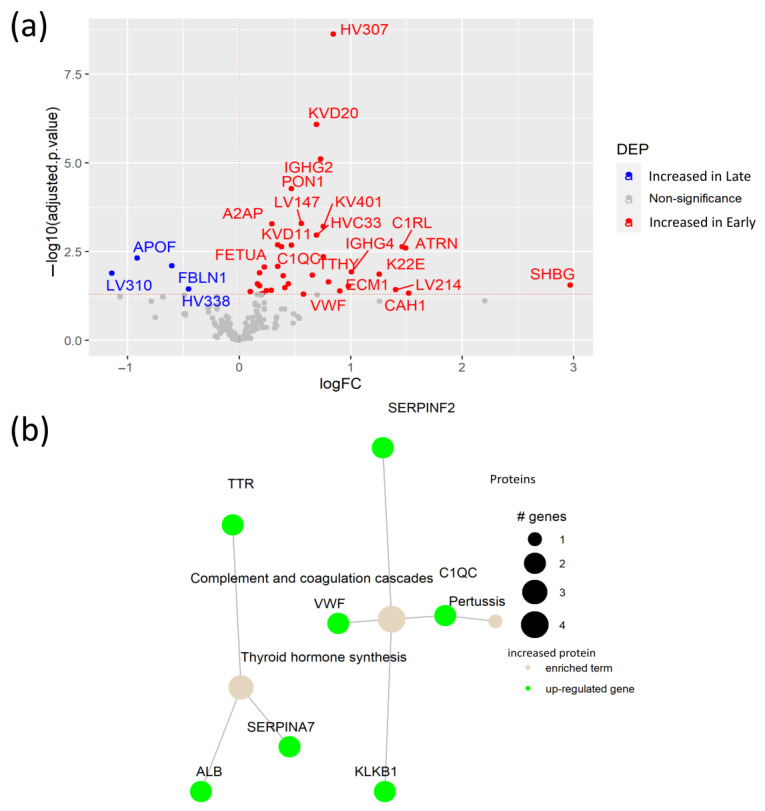
Plasma protein changes in early SARS-CoV-2 infection. (**a**) Volcano plot of DEPs between patients with early SARS-CoV-2 virus infection and patients with late infection; (**b**) Network of KEGG pathway enrichment analysis with DEPs via active subnetworks from STRING protein–protein interaction database.

To elucidate the plasma protein changes between early and late SARS-CoV-2 infection, we compared protein levels in samples collected between 14 early (<3 months after infection) and 14 late (>3 months after infection) plasma samples. This analysis revealed 36 proteins increased in patients at an early stage of the SARS-CoV-2 infection, including the antioxidant PON1, and four proteins with elevated levels in late infection (Figure 5a, Table S8). Interestingly, we detected increased levels of attractin (ATRN) in patients with early infection. ATRN is involved in the initial immune cell clustering during inflammatory responses, suggesting ATRN involvement in the initial response to the SARS-CoV-2 infection [32]. Moreover, elevated levels of keratin (K22E) and ECM1 in the plasma of patients during early infection indicate active tissue damage and remodeling due to the SARS-CoV-2 infection. In fact, ECM1 is a regulator of differentiation of several subsets of helper T cells, being a potential link between tissue damage and the immune response against the SARS-CoV-2 infection [33]. Pathway enrichment analysis showed an association between the elevated proteins in early infection, with the up-regulation of the complement and coagulation pathways, as well as thyroid hormone synthesis (Figure 5b, Table S9). Notably, although high levels of the Von Willebrand Factor (VWF) in COVID-19 patients were already reported [34], our study indicates that the VWF is specifically elevated in early SARS-CoV-2 infection. Additionally, increased A2AP was determined as a potential biomarker of early COVID-19 infection.

## 4. Discussion

Current understanding of COVID-19 pathophysiology needs further development to determine the role of the immune response against the SARS-CoV-2 infection and in the variability of clinical symptoms. Since the COVID-19 outbreak, several studies demonstrated that proteomics is a powerful tool for identifying biomarkers and characterizing COVID-19 pathophysiology [5,35,36,37,38,39]. For instance, the LC-MS/MS proteomics analysis of COVID-19 patients’ plasma combined with machine learning determined an 11-protein signature with a potential biomarker application and with a capacity to predict COVID-19 outcomes [35]. Another LC-MS/MS serum proteomics analysis determined the link between elevated IL-6 and the dysregulation of anti-microbial enzymes and coagulation factors in COVID-19 patients [36]. The longitudinal LC-MS/MS proteomics analysis of hospitalized COVID-19 patients and symptomatic control serum samples revealed an early decrease in innate immune proteins at the time of hospitalization with an increase in coagulation and lipid metabolism proteins [37]. Meanwhile, the longitudinal analysis of COVID-19 and symptomatic control plasma samples by Proximity Extension Assay (PEA) unveiled a protein signature associated with severity and organ damage [38]. Interestingly, another PEA study unveiled elevated proteins linked to COVID-19 severity, such as keratin (KRT)19, interleukin 1 Receptor-Like 1 (IL1RL1), TNF Receptor Superfamily Member 10b (TNFRSF10B), and V-Set and Immunoglobulin Domain Containing 4 (VSIG4). These protein signatures could differentiate three COVID-19 patient endotypes with distinct severity [39].

In this study, we performed an LC-MS/MS proteomics analysis of plasma from COVID-19 patients and their corresponding controls to characterize the systemic protein changes underlying the SARS-CoV-2 infection. As a result, changes in plasma levels of proteins involved in tissue damage and remodeling (K1C10, K22E, and ECM1), coagulation (FINC, F13A, ANGT, and the VWF), inflammation (A2AP, ZPI, and CBG), complement activation (C3, C1QC), and cholesterol metabolism (APOA1, APOL1, APOE, APOA4) as well as levels of antioxidant enzymes (PON1 and GPX3) were associated with the SARS-CoV-2 infection.

Our analysis detected for the first time the elevation of the keratins K1C10 and K22E in COVID-19 patients with pre-existing comorbidities, while previous studies found an enrichment of keratinization, increased plasma levels of KRT19 in COVID-19 patients, and association of KRT7 with COVID-19 severity [38,39,40]. Moreover, cytoskeletal remodeling of these keratins may be induced by the SARS-CoV-2 infection to facilitate the viral spread between epithelial cells, as K1C10 showed interaction with several SARS-CoV-2 proteins [41]. Additionally, these keratins are released with the cell content to the bloodstream and can be indicators of epithelial tissue damage [38]. Interestingly, K22E and ECM1 were also increased in early SARS-CoV-2 infection, making them potential candidates for diagnostic biomarkers of early SARS-CoV-2 infection. We confirmed that FINC is increased in COVID-19 patients despite the presence of pre-existing comorbidities, making it a potential biomarker of tissue damage by the SARS-CoV-2 infection [13].

Regardless of the comparison, pathway enrichment analysis revealed complement and coagulation cascade pathways to be upregulated in COVID-19 patients, which demonstrated their active role in the SARS-CoV-2 infection. Among the proteins involved in these enriched pathways, elevated C3 in COVID-19 patients with comorbidities was revealed. Previous reports demonstrated that C3 is associated with COVID-19 severity and was proposed as a potential treatment target to diminish inflammatory symptoms [26,42,43]. Meanwhile, the acute phase reactant A2AP was elevated in COVID-19 patients with comorbidities as well as in early infection. Interestingly, A2AP was also found elevated in persistent circulating plasma microclots of long-COVID-19 patients, highlighting A2AP’s contribution to the multiple coagulation/fibrinolysis pathophysiology of the SARS-CoV-2 infection [44]. In contrast, the negative acute-phase reactant CBG was found for the first time to be reduced in patients with SARS-CoV-2 and comorbidities. In fact, CBG has a role in dampening the excessive inflammatory response [17]. Similarly, the acute phase response protein ZPI was reduced in CPs compared to HCs. ZPI is increased in the acute phase response and promotes a reduction in the levels of inflammatory cytokines, such as interleukin (IL)-1, IL-6, and tumor necrosis factor- α (TNF-α) [16,45]. Recently, Toomer et al. showed that ZPI is increased in moderate and severe COVID-19 patients compared to healthy controls by ELISA [46]. These contradictory results could be due to differences in the analytical technologies used, as well as the different clinical characteristics, considering the presence of comorbidities in our cohort. The other two negative acute-phase reactants, FETUA and APOM, were reduced in COVID-19 patients without comorbidities, consistent with previous studies [27,28].

There are some controversies in the literature regarding ANGT levels in COVID-19 patients [47]. However, low levels of the blood regulator ANGT, together with high soluble ACE2 in severe COVID-19 patients compared to healthy subjects, were found in a previous study by LC-MS/MS [48]. Interestingly, our previous study also showed elevated levels of soluble ACE2 in the same cohort of COVID-19 patients with comorbidities [49]. These complementary results suggest that the SARS-CoV-2 infection alters the renin-angiotensin system, affecting the cardiovascular and inflammatory stability in patients with pre-existing comorbidities. Additionally, as ANGT is involved in stimulating TNF-α production and T cell activation, its reduction may negatively affect the adaptive immune response against SARS-CoV-2 [50].

Among the dysregulated metabolic enzymes, high levels of the antioxidant protein PON1 were found both in COVID-19 patients with comorbidities and patients with early infections in this study. Previous LC-MS/MS proteomics studies determined an increase in PON1 levels along the first month of infection, as well as a decrease in severity [30,37]. While our study and previous proteomics analysis measured the total PON1 protein abundance, another study showed that PON1 enzymatic activity is reduced in COVID-19, cancer, and obesity compared to healthy controls [51]. In contrast, GPX3 was decreased in COVID-19 patients with comorbidities—consistent with previous reports—which revealed lower glutathione peroxidase activity due to the SARS-CoV-2 infection [52] as well as Epstein–Barr virus infection in diabetic patients [53]. In fact, SARS-CoV-2 triggers oxidative stress, reinforcing inflammation and leading to a weakened antioxidant system, especially in patients with pre-existing comorbidities, generating oxidative stress [54].

The SARS-CoV-2 infection can cause biochemical modifications within erythrocytes that result in hemolysis. Consequently, patients develop anemia together with hyperferritinemia and systemic hypoxia, which can result in multi-organ failure [22]. Interestingly, HBB participates in the anti-viral innate immune response against RNA viruses [55]. Therefore, the SARS-CoV-2 induction of a defective HBB conformation may also block HBB anti-viral activity [22]. While previous studies demonstrated elevated levels of HBA and HBB in the airway mucus of severe COVID-19 patients as well as in the serum of patients with high IL-6 compared to healthy subjects [36,56], we also detected high plasma levels of HBA and HBB in patients with comorbidities compared to their disease controls.

The limitations of this study include a relatively low number of patients, and as they originate from a single center, the Finnish Clinical Biobank, it complicates the interpretation of results at the population level. Moreover, most patients were experiencing mild symptoms, which impeded the assessment of protein changes associated with COVID-19 severity. In addition to this, the LC-MS/MS proteomics analysis performance could be improved by using mass spectrometers with higher resolution in the Data Independent Acquisition (DIA) mode. Therefore, further validation in multi-center studies, including more and diverse ethnic groups, will facilitate the validation of the findings in specific comorbidities and the final application of the discovered novel biomarkers.

To conclude, this study determined a plasma protein signature shared by COVID-19 patients with pre-existing comorbidities characterized by alterations in acute-phase reactant proteins, coagulation and complement cascades, innate immune responses, as well as cholesterol and redox enzymes. Moreover, several potential biomarkers of early SARS-CoV-2 infection were identified; further research could establish their applicability in clinics.

## Data Availability

The data presented in this study is available in the Supplementary Data. The mass spectrometry proteomics data that support the findings of this study are available upon request to the corresponding author.

## Supplementary Materials

The following supporting information can be downloaded at https://www.mdpi.com/article/10.3390/biomedicines12040840/s1. Table S1: List of included SARS-CoV-2 infected patients in the study with their corresponding ICD-10 code in case of comorbidities, the sex-age-matched healthy and disease controls as well as the time of sample collection prior to 3 months; Table S2: List of differentially expressed proteins with UNIPROT identifier between patients with comorbidities and their healthy controls with the corresponding fold change as (patients with comorbidities (CP)—healthy controls (HC)) and the adjusted *p*-value; Table S3: List of KEGG enriched terms of differentially expressed proteins between patients with comorbidities and their healthy controls; Table S4: List of differentially expressed proteins with UNIPROT identifier between patients with comorbidities and their disease controls with the corresponding fold change as (patients with comorbidities (CP)—disease controls (DC)) and the adjusted p-value; Table S5: List of KEGG enriched terms of differentially expressed proteins between patients with comorbidities and their disease controls; Table S6: List of differentially expressed proteins with UNIPROT identifier between patients without comorbidities and their healthy controls with the corresponding fold change as (patients without comorbidities (NP)—healthy controls (HC)) and the adjusted p-value; Table S7: List of KEGG enriched terms of differentially expressed proteins between patients without comorbidities and their healthy controls; Table S8: List of differentially expressed proteins with UNIPROT identifier between patients with early versus late infection with the corresponding fold change as (patients with early infection (E)—patients with late infection (L)) and the adjusted *p*-value; Table S9: List of KEGG enriched terms of differentially expressed proteins between early and late SARS-CoV-2 infection.
